# Stockpiling Supplies for the Next Influenza Pandemic

**DOI:** 10.3201/eid1506.081196

**Published:** 2009-06

**Authors:** Lewis J. Radonovich, Paul D. Magalian, Mary Kay Hollingsworth, Gio Baracco

**Affiliations:** North Florida/South Georgia Veterans Health System, Gainesville, Florida, USA (L.J. Radonovich); Miami, Florida, USA (P.D. Magalian); North Florida/South Georgia Veterans Health System, Lake City, Florida, USA (M.K. Hollingsworth); Miami Veterans Affairs Healthcare System, Miami (G. Baracco)

**Keywords:** Influenza, pandemics, equipment, inventories, healthcare systems, mass casualties, emergency preparedness, bioterrorism and preparedness, online report

## Abstract

Faced with increasing concerns about the likelihood of an influenza pandemic, healthcare systems have been challenged to determine what specific medical supplies that should be procured and stockpiled as a component of preparedness. Despite publication of numerous pandemic planning recommendations, little or no specific guidance about the types of items and quantities of supplies needed has been available. The primary purpose of this report is to detail the approach of 1 healthcare system in building a cache of supplies to be used for patient care during the next influenza pandemic. These concepts may help guide the actions of other healthcare systems.

## Introduction and Historical Context

Preparations for the next influenza pandemic have captured a remarkable amount of attention, effort, and fiscal funding since 2004, when the scientific and public health communities became increasingly concerned about the emergence of a novel influenza virus (H5N1) infecting humans in Eurasia (*1*). Many feared the occurrence of an outbreak on the scale of the 1918–19 pandemic, during which one third of the world’s population became infected and as many as 100 million persons died (*2*)

Numerous guidance documents call for stockpiling certain supplies that might be needed to care for influenza patients during a pandemic (*3*–*6*). Just-in-time supply chains and standard operating procedures may be insufficient to meet demand as the number of cases increase (*3*–*6*). Healthcare systems have been challenged to determine the medical supplies that should be procured. Despite publication of numerous pandemic planning recommendations, little or no guidance has been available about this topic.

In December 2005, the US Department of Veterans Affairs (VA), which has governance over the largest integrated healthcare system in the United States, directed its medical centers to make detailed pandemic influenza preparations. This directive was ushered in by a guidance document (*7*) that broadly defined the goals and expectations of individual VA medical centers and provided a framework for planning and preparedness. Steps taken by medical centers, including decisions about stockpiling items, were determined by leaders at the local level.

To help our healthcare system prepare for a pandemic, a multidisciplinary group of experts drawn from within the VA system were empaneled to help bridge the gap between policy and procedure. Among the most challenging tasks was the development of a prioritized list detailing supplies and the essential quantities that should be stockpiled. This report aims to provide detailed example of 1 healthcare system’s approach to building a cache of supplies for the next influenza pandemic and to help identify critical gaps in knowledge that must be addressed for adequate preparedness.

## Steps toward Preparedeness

The 1,400 medical facilities in the national VA healthcare system are decentralized into 23 Veterans Integrated Service Networks (VISNs), each representing a specific region of the nation. The concepts described in this report are based on actions taken by staff in VISN 8, which includes southern Georgia, most of Florida, and all of Puerto Rico, and provides healthcare for ≈500,000 veterans. Regional network offices help integrate the activities of the medical facilities included in each VISN. Local medical center leaders are primarily responsible for the activities at each hospital and its affiliated outpatient clinics.

### Step 1: Committee Formation

The VISN 8 leadership appointed a multidisciplinary team to a pandemic influenza planning committee (PIPC) (Table 1) that was tasked to ensure a coordinated and consistent planning and response effort across the VISN. Although the committee had many responsibilities, the focus of this report is limited to the framework developed to estimate supply needs.

**Table 1:** Appointed members of the Pandemic Influenza Planning Committee*

Position	Areas of expertise
Hospital associate director†	Operational leadership
Physician	Infectious diseases; infection control
Physician	Biosecurity
Public affairs officer	Risk communication
Safety coordinator	Occupational hazards
Nurse practitioner	Infection control
Emergency planner	Emergency operations
Public safety director	Risk communication; safety regulation
Financial manager	Fiscal operations
Associate director of nursing	Nursing operations
Human resources manager	Human resources
Pharmacy manager	Pharmacy services; pharmacology
Education director	Employee education
Nurse educator	Staff and medical education
Safety director	Occupational and hospital hazards

*In addition, all members of the local Veterans Affairs medical center pandemic planning committees were invited to participate in the Pandemic Influenza Planning Committee. †Chair.

The PIPC agreed on a set of basic philosophical principles that guided our pandemic preparation efforts. During a pandemic, the first priority would be to provide the best possible care to patients while maximizing healthcare worker safety. Essential and relatively affordable patient care supplies and medications meant for basic life support (e.g., intravenous fluids, oxygen, and antimicrobial drugs) would be purchased first, and more expensive, technologically advanced life support (e.g., mechanical ventilation) equipment would be purchased when additional funds become available. This approach to balancing utilitarian and deontologic decision making is discussed elsewhere (*8*,*9*). Vaccines and antiviral drugs would not be relied upon as primary means of intervention because their availability and effectiveness during a pandemic remain uncertain (*4*,*10*). Although plans to acquire, store, distribute, and administer these countermeasure supplies would be made when possible and necessary, we acknowledge that these plans would not be relied upon as primary countermeasures in most pandemic scenarios.

### Step 2: Agreeing on Assumptions to Key Questions

PIPC members recognized that the uncertainty surrounding a pandemic would require a series of assumptions and that any assumption would include some guesswork. To minimize errors, available historical data and guidance from governmental institutions were used to estimate the effect on our healthcare system.

#### How Many Persons Should We Expect Would Seek Healthcare at Our Facilities?

Most tools estimate effect on healthcare facilities based on population size, but we were dealing with a subpopulation of veterans that may seek care at the VA facilities or at any other community resource. In addition, VA facilities may open their doors to nonveterans during a pandemic. We decided, arbitrarily, to define our universe of patients as the number of individually enrolled persons who sought care at VA facilities during the previous fiscal year. This figure enabled us to calculate system and facility needs in a standardized fashion.

Once the number of patients was established, we used the US Department of Health and Human Services (DHHS) 1918-scale pandemic model (*11*) (Table 2) and FluSurge version 2.0 software (*12*) (Table 2) to estimate the number of persons who would be expected to seek care, be hospitalized, admitted to an intensive care unit (ICU), or be treated with mechanical ventilation (Table 2). The only modification to the DHHS model was in the proportion of the population likely to contract influenza. The model calls for 40% disease incidence for children, 20% for healthy adults, and a somewhat higher incidence for elderly persons. Therefore, 25% seemed like a reasonable number for the VA, an institution that does not provide healthcare to children. We based calculations on the population likely to request care, not on the physical or personnel capacity of our facilities. It was our assertion that physical capacity would be increased and standards of healthcare would be lowered, as necessary, during a pandemic to permit serving as many people as possible. We acknowledge that alternate sites of care might become available during a pandemic. However, we viewed this possibility as too unpredictable to include in our assumption model.

**Table 2:** Pandemic influenza surge assumptions and calculations per wave for a 1918-like influenza pandemic*

Assumption/calculation	Value
US Department of Health and Human Services assumptions	
Total population	300,000,000
No. ill	90,000,000
No. with outpatient medical care	45,000,000
No. hospitalized	9,900,000
No. needing ICU	1,485,000
No. needing mechanical ventilation	745,500
No. deaths	1,903,000
FluSurge assumptions	
Average length of non-ICU hospital stay for influenza-related illness, d	5
Average length of ICU stay for influenza-related illness, d	10
Average length of ventilator use for influenza-related illness, d	10
Average % of admitted influenza patients who need ICU care	15.0
Average % of admitted influenza patients who need ventilators	7.5
Average % of influenza deaths assumed to be hospitalized	70.0
Vital statistics calculations	
Attack rate†	30.0
% Ill treated as outpatients	50.0
% Seeking outpatient care treated as inpatient	22.0
% Hospitalized treated in ICU	15.0
% Hospitalized needing mechanical ventilation	7.5
Influenza-associated case-fatality rate (per 100 cases)	2.1

*Adapted from US Health and Human Services pandemic planning assumptions (www.pandemicflu.gov/plan/pandplan.html). ICU, intensive care unit. †Attack rate, % of persons infected by influenza in a community (assumed to be constant from community to community in our catchment area).

#### What Length of Hospital Stay Would Be Required by Our Patients?

Length-of-stay figures were needed to calculate supply needs because resource use is more accurately calculated by patient-days of care instead of number of admissions. We used some of the assumptions made by FluSurge version 2.0 as follows: average length of stay (not in ICU) of 5 days per patient, an additional 10 days for those requiring an ICU stay, and an average time receiving mechanical ventilation of 10 days.

#### What Personal Protective Equipment Would Be Needed To Care for Patients with Pandemic Influenza?

Among the gaps in knowledge regarding pandemic influenza is the mechanism of human-to-human transmission of influenza (*10*). The Institute of Medicine recommendation (*10*) to consider all transmission routes probable and consequential was accepted. Precautions against standard, contact, droplet, and airborne transmission (*13*) were incorporated into the plan. We assumed that sole use of disposable N95 respirators would be prohibitively expensive or otherwise not possible because of global shortages (*14*). Instead, we decided that staff with prolonged periods of exposure (e.g., physicians, nurses, respiratory technicians, selected housekeepers) would be issued and that just-in-time fit testing, a reusable elastomeric half-face mask with 3 sets of filters, would be used. We estimated that we would need ≈1,000 of these masks and reusable goggles for each 50,000 patients served (on the basis of the size and catchment population of one of our medium-size facilities). Disposable masks would be limited to the beginning of the pandemic and to personnel with infrequent exposure. Using these principles, we calculated the workload, supplies, and medication required to care for typical influenza patients. Accordingly, estimates were produced for the average needs of influenza patients requiring >1 types of services, including outpatient, inpatient medical ward, or ICU settings with or without mechanical ventilation.

### Step 3: Calculating Supply and Medication Needs

We estimated the per patient–encounter needs by staff category (Table 3) and the number of healthcare worker contacts per patient, per day, for each type of healthcare setting (Table 4). In a similar fashion, supply needs were estimated per patient encounter (for outpatients) or per patient-day of care (for inpatients) (Table 3). The ascertained supply and medication needs were combined in a spreadsheet to estimate the needs of each facility and for our network. Spreadsheet formulas enabled the needs of each facility or healthcare system to be easily modified by using the number of individually enrolled patients (Table 5).

**Table 3:** Personal protective equipment needs by staff category during a 1918-like influenza pandemic

Staff with no or infrequent patient exposure and/or direct contact (e.g., fiscal personnel)
1 disposable N95 respirator per exposure or contact
1 disposable gown per contact
1 pair of disposable gloves per contact
Staff with prolonged periods of exposure and/or direct patient contact (e.g., nurses, physicians)
1 reusable* N95 respirator per outbreak
1 pair of goggles per outbreak
1 pair of disposable gloves per contact
1 disposable gown per contact
2 disposable respirator cartridges per month
Staff with prolonged periods of exposure but no or infrequent direct contact with patients (e.g., emergency department clerk)
1 reusable† N95 respirator per outbreak
1 pair of goggles per outbreak
1 disposable gown per shift
1 pair of disposable gloves per contact
2 disposable respirator cartridges per month

*Recent reports (Institute of Medicine and Office of Health Safety [*14*]) and the Occupational Health and Safety Administration [*6*]) indicate that there is a strong likelihood of disposable respirator shortages during an influenza pandemic. Reusable respirators were selected as the primary component of respiratory protection for healthcare workers to avoid the possibility of exhausting local supplies of disposable respirators. †Disposable N95 respirators may need to be used during early stages to enable time for fit-testing and issuing of reusable masks to pertinent staff.

**Table 4:** Estimated personnel contacts and supply requirement assumptions for each patient during a 1918-like influenza pandemic*

Type of patient	Personnel contacts			Supplies	
	Type	Quantity		Category	Quantity
Outpatients in ED, UC, or PC clinic not requiring admission	P or NP	1		Disposable medical masks	1
	N	2		Home care kit†	1
	SW or MHC	1			
	A	1			
Outpatients in ED, UC, or PC clinic requiring admission	P or NP	3		Disposable medical masks	1
	N	5		Oxygen, NC	1
	RadT	1		IVF (1-L bag)	1
	A	2		IV kit	1
	RespT	3		Antipyretic drugs, DDD	1
	E	1		Antimicrobial drugs‡, DDD	1
				Antiviral drugs, DDD	1
Inpatients in general ward (non-ICU) per day	P or NP	2		Disposable medical mask	0§
	N	6		Oxygen, NC or FM	1
	RespT	6		Antipyretic drugs, DDD	4
	SW or MHC	1		Antimicrobial drugs‡, DDD	1–3
	RadT	1		Antiviral drugs, DDD	3
	Ptech	1		IVF (1-L bag)	3
	HK	1		IV kit	1
Inpatients in ICU not receiving mechanical ventilator per day	P or NP	4		Oxygen, NC, or FM	1
	N	24		Albuterol nebulization with masks	6
	RespT	12		Antipyretic drugs, DDD	4
	SW or MHC	1		Antimicrobial drugs¶, DDD	1
	RadT	2		Antiviral drugs, DDD	3
	HK	1		IVF (1-L bag)	3
				IV kit	1
				Blood gas kits	3
Inpatients in ICU receiving mechanical ventilator per day	P or NP	4		Oxygen, FM, or NRB	1
	N	24		IVF (1-L bag)	3
	RespT	6		IV kit	1
	SW or MHC	1		Ventilator circuit and filters	0.2
	RadT	2		Antipyretic drugs, DDD	4
	HK	1		Antimicrobial drugs¶, DDD	1
				Antiviral drugs, DDD	3
				Suction kit	1
				Midazolam plus ropofol, DDD	1
				Vasopressors, DDD	1
				Proton pump inhibitors	1
				Morphine sulfate, DDD	5
				Albuterol nebulization units	6
				Central line kit	1

*ED, emergency department; UC, urgent care; PC, primary care; P, physician; NP, nurse practitioner; N, nurse; SW, social worker; MHC, mental health counselor (or clergy); A, administrative (including clerks); NC, nasal cannula; RadT, radiology technician; IVF, intravenous fluids; IV, intravenous; DDD, defined daily dose; RespT, respiratory therapist; E, escort; ICU, intensive care unit; FM, face mask; Ptech, phlebotomy technician; HK, housekeeper; NRB, nonrebreather mask. †A home care kit includes (VHA pan flu plan, Appendix D-4) thermometers, nonsteroidal antiinflammatory drugs or acetaminophen, cough suppressants, oral rehydration mix packs, a medical mask, printed home instructions about caring for someone with influenza, a list of contact information, and a list of signs or symptoms that should prompt a call or visit to a healthcare center. ‡Ceftriaxone plus azithromycin, or moxifloxacin. §Provided in ED (1 per admission). ¶Vancomycin plus piperacillin/tazobactam.

**Table 5:** Formulas, prioritization, and example calculations of supplies for a 1918-like influenza pandemic*

Assigned variable name†	Item‡	Formula	Priority	Example calculation (patient population = 500,000)	Comments and explanation of calculation
Vital statistics					
B1	Fiscal year 2005 unique enrollees	B1	500,000		
B2	Illness	B2 = B1 × 0.25	125,000	B2 = B1 × 25% attack rate	
B3	Seek healthcare	B3 = B2 × 0.5	62,500	B3 = B2 × 50% of ill seek healthcare	
B4	Hospitalized	B4 = B3 × 0.22	13,750	B4 = B3 × 22% hospitalized	
B5	ICU care	B5 = B4 × 0.15	2,063	B5 = B4 × 15% treated in ICU	
B6	Mechanical ventilation	B6 = B5 × 0.5	1,032	B6 = B5 × 50% receive mechanical ventilation	
B7	Deaths	B7 = B4 × 0.25	3,438	B7 = B4 × 25% case-fatality rate among hospitalized	
B8	Outpatient visits, not hospitalized	B8 = B3 – B4	48,750	B8 = those seeking healthcare not hospitalized	
B9	Hospitalized patient days	B9 = B4 × 5	68,750	B9 = B4 × 5 d average length of stay	
B10	ICU patient-days (not receiving mechanical ventilation)	B10 = B5 × 0.5 × 10	10,315	B10 = B5 × 50% no mechanical ventilation × 10 d average length of stay	
B11	ICU mechanical-ventilation days	B11 = B6 × 10	10,315	B11 = B6 × 10 d average length of stay	
Contacts					
C1	Physician	C1 = B8 + (B4 × 3) + (B9 × 2) + (B10 × 4) + (B11 × 4)	310,020	C1 = no. not hospitalized + 1 physician contact (from Table 4) + no. hospitalized × 3 physician contacts (from Table 4)	
C2	Nurse	C2 = (B8 × 2) + (B4 × 5) + (B9 × 6) + (B10 × 24) + (B11 × 24)	1,073,870		
C3	Respiratory technician	C3 = (B4 × 3) + (B9 × 6) + (B10 × 12) + (B11 × 6)	639,420		
C4	Radiology technician	C4 = B4 + B9 + (B10 × 2) + (B11 × 2)	123,760		
C5	Phlebotomist	C5 = B9	68,750		
C6	Housekeeper	C6 = B9 + B10 + B11+ 1,000	90,380		
C7	Other HCW (mental health, clergy)	C7 = B8 + B9 + B10 + B11	138,130		
C8	Administrative	C8 = B9 + B4 × 2	96,250		
C9	Escort	C9 = B4	13,750		
Personal protective equipment					
D1	Gloves	D1= (C1 + C2 + C3 + C4 + C5 + C6 + C7 + C8 + C9) × 2 × 1.2	A	6,130,392	D1 = (physician contacts + nursing contacts+ escort contacts) × 2 gloves per pair × 120% to account for unforeseen needs
D2	Gowns	D2 = (C1 + C2 + C3 + C4 + C5 + C6 + C7 + C8 + C9) × 1.2	A	3,065,196	
D3	N95 disposable respirators	D3 = C8 + C9 +10,000 × B1/50,000	A	210,000	D3 = administrative contacts + escort contacts + an extra 10,000 respirators per 50,000 patients to account for unexpected or unforeseen needs
D4	Goggles	D4 = 1,000 × B1/50,000	A	10,000	D5 = 1,000 respirators per 50,000 patients
D5	Reusable respirators	D5 = 1, 000 × B1/50,000	A	10,000	See D5
D6	Reusable respirator filter cartridges	D6 = D5 × 3	A	30,000	
D7	Disposable mask (for patients)	D7 = (B8 + B4) × 1.2	A	75,000	
Essential supplies					
E1	Oxygen nasal cannulas	E1 = B4	A	13,750	
E2	Oxygen masks	E2 = B4 + B10	B	24,065	
E3	Nonrebreather masks	E3 = B10	A	10,315	
E4	Circulaire nebulizers	E4 = B4 + B5	B	15,813	E3 = 1 nebulizer for each patient who is hospitalized and each patient who requires ICU care (each considered separately)
E5	Circulaire masks/filters	E5 = B4 + B5	B	15,813	
E6	IV tubing	E6 = B4 + (B9 × 2) + (B10 × 4) + (B11 × 4)	A	233,770	
E7	Heparin lock kits	E7 = B4 + (B9 × 0.33) + (B10 × 0.33) + (B11 × 0.33)	A	43,245	
E8	Central line kits	E8 = B5	B	2,063	
E9	Blood gas kit	E9 = (B10 × 3) + (B11 × 3) + B9	B	130,640	E9 = ICU without mechanical ventilation patient-days × 3 kits per ICU stay + ICU mechanical ventilation-days × 3 kits per stay × 1 kit per non-ICU stay
E10	Suction kit	E10 = B11	A	10,315	
E11	Ambulance bags	E11 = B6	A	1,032	
E12	Alcohol-based hand cleaners	E12 = (C1 + C2 + C3 + C4 + C5 + C6 + C7 + C8 + C9) × 0.01	A	25,543	
E13	Disposable ventilators	E13 = 60 × B1/50,000	C	600	E13 = 60 ventilators per 50,000 patients
E14	Ventilator supplies	E14 = B6	B	1,032	
E15	Morgue packs	E15 = B7	B	3,438	
Medication					
F1	Home care kits	F1 = B8	B	48,750	
F2	IV fluids: D5NS (1-L bags)	F2 = (B4 × 2) + (B9 × 3) + (B10 × 4) + (B11 × 4)	A	316,270	
F3	Antipyretics (in DDD)	F3 = B4 + B9 + B10 + B11	B	103,130	F3 = non-ICU patient days + ICU without mechanical ventilation patient-days + ICU mechanical ventilation patient-days in daily-dose equivalents
F4	Ceftriaxone (in DDD)	F4 = B4 + B9 × 0.75	A	65,313	Arbitrarily assume that among those who are treated initially with ceftriaxone and azithromycin, 75% will continue on the same regimen and 25% will be switched to moxifloxacin
F5	Azithromycin (in DDD)	F5 = B4 + B9 × 0.75	A	65,313	
F6	Moxifloxacin (in DDD)	F6 = B9 × 0.25	B	17,188	
F7	Vancomycin (in DDD)	F7 = B10 + B11	B	20,630	
F8	Piperacillin/tazobactam (in DDD)	F8 = B10 + B11	B	20,630	
F9	Albuterol unit doses	F9 = (B9 × 4) + (B10 × 6) + (B11 × 6)	B	398,780	
F10	Sedation (midazolam or propofol in DDD)	F10 = B11	B	10,315	
F11	Norepinephrine (in DDD)	F11 = B11	C	10,315	
F12	Proton pump inhibitors, IV, (in DDD)	F12 = B11	C	10,315	
F13	Morphine (in DDD)	F13 = B11	B	10,315	

*ICU, intensive care unit; HCW; healthcare worker; IV, intravenous; D5NS, KCl in 5% dextrose and NaCl; DDD, daily defined dose. †To understand this table and use the calculations for a selected population of patients, view the format like a table with the assigned variable name representing the name given to the figure (the variable) that is produced when calculating the figure in that row. For example, the figure that is produced from calculating B4 (13,750) is the number of patients who can be expected to be hospitalized among a patient population of 500,000. In turn, to arrive at the figure for B5 (the number who require ICU care) the figure that was produced for B4 (13,750) is multiplied by the 15% treated in the ICU. Thus, the definition of B5 (2,063) is the number of patients who require ICU care, which is calculated (and then assumed) to be 15% of the patients who are hospitalized. ‡Not necessarily an exhaustive list; additional items may be added in the same fashion.

### Step 4: Prioritizing Supply Needs

Because limited financial resources were available, the PIPC was asked to establish a prioritization scheme. Although every item on the list was considered important, each was subcategorized into purchase priority A, B, and C; A was the most important (Table 5). To arrive at the category level, the following scheme was used. Category A was personal protective equipment, basic life-support items (intravenous fluids, oxygen), and first-line antimicrobial drugs. Category B was second-line antimicrobial drugs, ventilator supplies, sedatives, nebulizers and β-agonists, home care packs, and morgue packs. Category C was disposable ventilators, proton pump inhibitors, and vasopressors. Antiviral medications and vaccines were not included in this list because it was expected that the VA would acquire and maintain a centralized cache of oseltamivir, and vaccine availability and effectiveness were unknown.

### Step 5: Compromising

The calculated cost of purchasing all essential items for a population of 500,000 amounted to ≈$11 million. Despite efforts to prioritize the items into 3 categories, the calculated cost of category A items far exceeded the amount of funds available. The PIPC debated the best approach and recommended that the available funds be used to purchase a percentage of category A items and that future funds would be used to purchase additional category A items and decreasing percentages of category B and category C items. For example, the funds available at that time were sufficient to purchase 12.5% of category A items. Upon the availability of future funding, perhaps an additional 7.5% of category A items would be purchased along with 5% of category B items and 2.5% of category C items.

### Step 6: Ordering Items

Purchasing items in large quantity through a prenegotiated agreement enabled a discount off retail prices. However, despite this contract, back-order delays occurred (and would be expected to occur during a pandemic) for several key items. One supplier of personal protective equipment indicated that shipment would be delayed by 6–9 months, affirming predictions of shortages of personal protective equipment. This experience underscored making purchases well in advance of the date when the items were expected to be used for patient care.

### Step 7: Storing Items

Storage of supplies proved to be among the most resource-intensive components of cache-building. Although initial wishes were to store a cache on the campus of each medical center, the space necessary was too large for most VA institutions to accommodate. After extensive discussion and careful analysis of options, a decision was made to store pandemic supplies in a 10,000–square foot, temperature-controlled, leased warehouse. Quoted costs for space ranged from $10 to $14 per square foot per year ($100,000–$140,000 per year for 10,000 square feet). The recommended location was near an airport to ensure efficient transport of supplies either by tractor trailer or by air cargo. A back-up emergency generator was included to maintain air-conditioning in the event of a power failure.

Many items purchased for the cache had expiration dates. Although most items had multiple-year shelf lives, some shelf lives were as short as 1 year. The variability of manufacturer-ascribed expiration dates and other reasons for supply rotation led to the recognition that the cache would become a dynamic component of medical system supplies. Items would need to be inspected regularly and rotated through the storage facility on a regular basis. To meet this need, a human resource commitment of 1 full-time employee equivalent would be necessary for logistics management of the inventory. Duties of this person would include inspecting the inventory, assisting with incoming and outgoing deliveries, rotating items into the routinely used supplies of the medical system to ensure use before expiration, and prodding physical security for the inventory. This person would also be charged with developing and maintaining a plan for transportation and deployment of the inventory in the event of a pandemic. In addition, each medical facility would also be required to provide an employee to help manage the inventory and who would report to the cache in the event of a pandemic.

## Discussion and Recommendations

Despite the numerous uncertainties posed by pandemic influenza, the types and quantities of essential items that should be stockpiled can be estimated by using a reasoned approach. What is offered in this report is a method to calculate the components of a stockpile by using assumptions that are drawn from previous pandemics. This method enables modification of figures, making them scalable and adaptable to any size population. By following the logic of the proposed calculations, it should be possible to modify the assumptions and other figures as needed for almost any community or healthcare system.

Perhaps the most important limitation with this method is a reliance on assumptions. No one knows what the next pandemic will bring. We believe that it is better to plan for a more severe event that will leave the system overprepared than to risk being underprepared. However, this approach may be viewed by some as unnecessary or too expensive. A stratified purchase plan, in which a fraction of essential items is purchased periodically, is recommended on the basis of availability of funds. Some may favor purchase of all items in 1 category before moving to the next category.

The formation and management of a pandemic supply cache would require considerable human and financial resources. The level of commitment may be viewed by some healthcare systems as too costly, especially in an era of economic instability and healthcare system instability likely requiring major reform. Some of the more resource-intensive components of the proposed approach, such as the storage of items in a staffed central facility, were facilitated by the large size of our healthcare system and available resources. Achieving a similar product in the private sector, where healthcare systems are typically much smaller than those in the federally managed VA, might require a partnership among multiple healthcare systems in a region.

One of the most common and widely held misconceptions we encountered was the notion that a healthcare system could be stressed to the breaking point, such that a large surge of patients could eventually render a hospital unable to function. It is our view that in reality, healthcare systems are designed to operate in a graded fashion. Although it is theoretically conceivable that a catastrophe could cause hospitals to cease functioning, it is much more likely that they will continue functioning, even under the most ominous circumstances (*15*–*17*). What will change is the standard of care that is delivered; many patients may have access to less resources than would normally be available (*18*,*19*). Stockpiling supplies should help prepare for a downgraded level of care that becomes inevitable as resources are increasingly stretched. A key message taken from our experiences was that supplies need to be ordered far in advance of a pandemic to avoid major problems with back orders and supply shortfalls.

The estimated cost of purchasing all supplies and medications needed to provide healthcare to a population of 500,000 during a wave of an influenza pandemic, including negotiated and contracted prices, was ≈$11 million, or approximately half that amount if one only considers purchasing priority A items. This amount is considerably higher than the amount estimated elsewhere (*20*). This difference may stem from the way we calculated our needs: we did not assume that we could reach a full capacity. Instead, we attempted to estimate the population that is likely to seek care and assumed that under the dire circumstances of a severe pandemic facilities would decrease standards of care, open alternate sites of care, and creatively care for those patients who came for treatment. We also did not assume that we would have a shortage of personnel to care for these patients. Through altered standards of care; emergency privileging and cross-training of healthcare workers, volunteers, and other persons willing and able to care for our patients, a temporary and substantially changed workforce would be expected to emerge.

Numerous gaps in knowledge were encountered. The mechanisms of human-to-human transmission of seasonal and pandemic influenza are poorly understood. Numerous articles have discussed the types of respiratory protection that should be considered and stockpiled (*6*,*10*) for healthcare workers. However, there has never been a definitive, prospective clinical trial that shows whether respirators are superior to surgical masks. Translational research funding to answer these questions should become a priority.

This report is an attempt to describe the challenges our healthcare system faced when preparing for an influenza pandemic. By no means are all the answers, or even the questions, reported here. Additional work is needed to further identify important questions and appropriate solutions. We hope these concepts will help guide the decisions of other healthcare systems as they work through this challenging task.
